# Safety and Feasibility of a Fast-Track Pathway for Neurosurgical Craniotomy Patients: Bypassing the Intensive Care Unit

**DOI:** 10.1016/j.mayocpiqo.2023.09.002

**Published:** 2023-11-15

**Authors:** Carlos Perez-Vega, Devang K. Sanghavi, Pablo Moreno Franco, Ryan M. Chadha, Alberto E. Ardon, Elird Bojaxhi, Klaus D. Torp, Lisa A. Marshall, Tiffany M. Halstead, Valentino E. Ford, Lynda M. Christel, Sanjeet S. Grewal, Kaisorn L. Chaichana, Alfredo Quinones-Hinojosa, Levi W. Howard, W. Christopher Fox, William D. Freeman, NPCU Group, Lesia H. Mooney, Daniel J. Jerreld, Karen G. Waters, Greg Coltvet, Eric W. Nottmeier, Josephine F. Huang

**Affiliations:** a Department of Neurologic Surgery, Mayo Clinic, Jacksonville, FL; b Department of Critical Care Medicine, Mayo Clinic, Jacksonville, FL; c Department of Anesthesiology, Mayo Clinic, Jacksonville, FL; d Department of Nursing, Mayo Clinic, Jacksonville, FL; e Department of Neurology, Mayo Clinic, Jacksonville, FL

## Abstract

**Objective:**

To describe the safety and feasibility of a fast-track pathway for neurosurgical craniotomy patients receiving care in a neurosciences progressive care unit (NPCU).

**Patients and Methods:**

Traditionally, most craniotomy patients are admitted to the neurosciences intensive care unit (NSICU) for postoperative follow-up. Decreased availability of NSICU beds during the coronavirus disease-2019 delta surge led our team to establish a de-novo NPCU to preserve capacity for patients requiring high level of care and would bypass routine NSICU admissions. Patients were selected a priori by treating neurosurgeons on the basis of the potential need for high-level ICU services. After operation, selected patients were transferred to the postoperative care unit, where suitability for NPCU transfer was reassessed with checklist-criteria. This process was continued after the delta surge.

**Results:**

From July 1, 2021 to September 30, 2022, 57 patients followed the NPCU protocol. Thirty-four (59.6%) were women, and the mean age was 56 years. Fifty-seven craniotomies for 34 intra-axial and 23 extra-axial lesions were performed. After assessment and application of the checklist-criteria, 55 (96.5%) were transferred to NPCU, and only 2 (3.5%) were transferred to ICU. All 55 patients followed in NPCU had good safety outcomes without requiring NSICU transfer. This saved $143,000 and led to 55 additional ICU beds for emergent admissions.

**Conclusion:**

This fast-track craniotomy protocol provides early experience that a surgeon-selected group of patients may be suitably monitored outside the traditional NSICU. This system has the potential to reduce overall health care expenses, increase capacity for NSICU bed availability, and change the paradigm of NSICU admission.

The coronavirus disease-2019 (COVID-19) pandemic imposed many challenges on health care systems by causing rapid changes in hospital censuses, workflows, staffing, personal protective equipment (PPE) shortages, and other workflow changes. With newer COVID-19 variants and possible waves of patients needing mechanical ventilation, ICU shortages made it challenging to treat patients in need of neurosurgical intervention and needing an ICU bed.^1^, ^2^, ^3^

The standard of care for most craniotomy patients at most neurological or neurosurgical centers is to admit them to the neurosciences intensive care unit (NSICU) with nursing that can detect potential complications, such as intracranial hemorrhage, seizure activity, infection, and more.^4^^,^^5^ Most of these craniotomy patients spend 1 to 2 days in the NSICU and are later transferred, once stable neurologically, to a regular hospital bed before being discharged home or to a rehabilitation facility. We hypothesized that the neurosurgeon can select patients who have a lower risk of needing ICU level interventions such as prolonged intubation or mechanical ventilation and predict the need for external ventricular drain placement during the preoperative and intraoperative period.

During the COVID-19 delta surge, we worked with institutional leaders to select a priori a subset of lower risk neurosurgery patients to create an intermediate bed strategy called neurosciences progressive care unit (NPCU). The NPCU was defined as intermediate level of care between NSICU level of care and standard hospital bed management. On the basis of this idea, we present our preliminary experience with the safety and feasibility of neurosurgical craniotomy patients being managed in an NPCU postoperatively.

## Methods

### Study Design and Background

During July 1, 2021 to October 31, 2021, a single tertiary care academic hospital experienced an unprecedented demand for hospital beds above our licensed capacity because of the number of COVID-19 delta patients requiring hospitalization. In response, the surgical schedule was decreased, and elective procedures were postponed to a later date unless there was a pressing medical urgent or emergent need. To accommodate the surge in COVID-19 patient capacity, the anesthesia team converted our 50-bed postoperative care unit (PACU) on the fourth floor of the hospital into a COVID overflow unit. A smaller PACU on the fifth floor used for previous gastrointestinal endoscopy procedures was then converted into a new postoperative PACU. Within this new workflow, the neurology or neurosurgery practice had patients that could not wait for elective or deferred operation and had urgent (eg, brain tumor with mass effect and aneurysms) or emergency conditions (eg, acute stroke). This led our institutional leaders to create an administrative NPCU protocol whereby a subgroup of patients with an overall lower risk of requiring high-level intensive care services would be monitored postoperatively by the NSICU team, assuming there was no need for intubation or mechanical ventilation or external ventricular drains. This NPCU model was considered by leaders as an alternative and temporary strategy for neurosurgery overflow because of a rapid shortage in ICU beds during the COVID-19 delta surge. Craniotomy patients traditionally admitted to the NSICU but instead admitted to this new NPCU protocol were included in this study.

Patients admitted to this NPCU after the surge from November 1, 2021 to September 30, 2022, were included in the analysis. Because the model was considered an administrative review of standard of care and reviewed as part of ongoing patient care quality and safety efforts, this retrospective analysis was deemed exempt from institutional review board review, and patient consent was not required.

### The Neurosciences Progressive Care Unit Concept

In 2018, we reported a stroke PCU model at our institution for thrombolysis patients with similar neurology nurse staffing ratios, but it did not accommodate neurologic operation pateints.^6^ On the basis of our previous experience, we therefore designed this model for neurosurgical patients undergoing intracranial tumor resection. The NPCU was located on the same fifth floor of our hospital as the neurology or stroke PCU. However, to make the NPCU ICU-monitoring capable before launch, we installed Phillips-ICU monitors on each bed, which allowed viewing of vital signs remotely through our electronic medical record (Epic) on a large liquid-crystal display monitor within our NSICU and was 1 floor below the fifth floor NPCU. Therefore, these NPCU patients were managed by the NSICU team primarily. We installed a telemedicine robot (TeleDoc) system (RP7-lite) and multiple TeleDoc tablets on rolling stands to augment nursing support and patient monitoring on a large liquid-crystal display screen within a centralized NSICU office within a fourth floor NSICU command center.

### Criteria for Admission to NPCU and Clinical Outcomes

**Figure 1:**
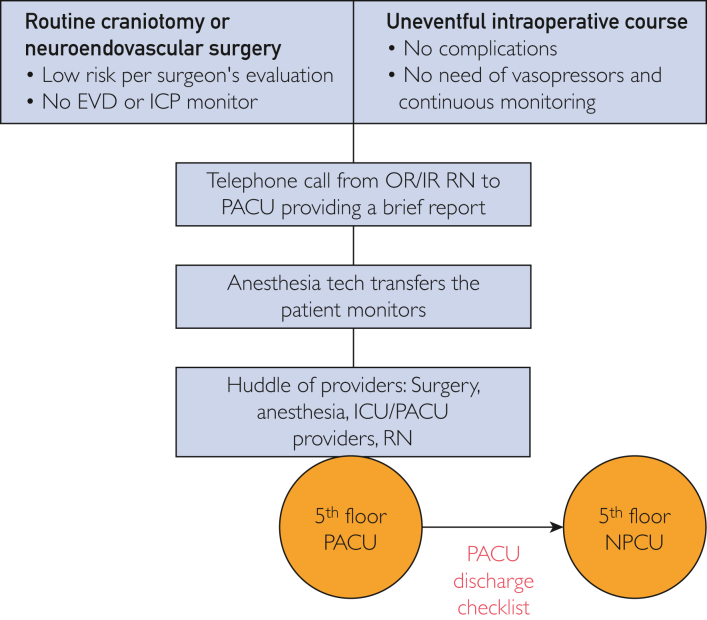
NPCU patient selection protocol diagram. Patients were selected a priori by the treating surgeon (top of diagram) and reassessed after admission to PACU by the anesthesia team in conjunction with neurocritical care attending and team. EVD, external ventricular drain; ICP, intracranial pressure; IR, interventional radiology; NPCU, neurosciences progressive care unit; OR, operative room; PACU, postoperative care unit; RN, registered nurse.

The subgroup of patients admitted to the NPCU was selected a priori by the treating tumor neurosurgeon on the basis of the potential need for high-level ICU services, such as vasopressor or antihypertensive drips, airway failure or intubation or mechanical ventilation, and invasive brain devices (eg, external ventriculostomy). After operation, patients were transferred to the fifth floor PACU, where they were observed for up to 2 hours and evaluated by anesthesia, neurocritical care, neurosurgery, and nursing teams for suitability to transfer to NPCU. The checklist-criteria included the following: no immediate postoperative complications (intracranial hemorrhage, seizure episode), no intracranial pressure monitor or external ventricular drain placement, no need of volume resuscitation or vasopressors, no need for invasive ventilation, and no signs of clinical deterioration (Table 1). If 1 of these criteria were not met, the patient would be transferred to NSICU. Once in the NPCU, the neurocritical care team oversaw rounding and treatment plans, and neurological checks were performed hourly during the first 12 hours, then downgraded to routine floor evaluations every 4 hours thereafter, and care would later be handed off to the neurosurgical floor team if the patient remained clinically stable or progressing (eg, able to tolerate oral administration, or get out of bed with physical therapy) (Figure 1); different than a formal NSICU admission, where a patient would remain in hourly neurological checks for at least 24 hours and until stopped, with a later transfer of the patient to a different floor.

**Table 1:** Criteria for NPCU Admission by Anesthesia and Neurocritical Care Teams

Postoperative Criteria for NPCU Admission
No immediate postoperative complications (intracranial hemorrhage or seizure)
No intracranial pressure monitors or external ventricular drain placement
No need of volume resuscitation or vasopressors
Absence of signs suggesting clinical deterioration

NPCU, neurosciences progressive care unit.

Collected variables included sex, age, diagnosis, type of intervention, length of stay, need of transfer to NSICU, readmission rate, and overall expected clinical outcome for a disease. We defined the primary safety metrics as a diversion to intensive care floor space after the patient was placed in the NPCU, other patient care events, and standard outcomes such as length of stay and overall clinical outcome. Feasibility was defined as the ability to predict the accurate placement of these patients preoperatively into the NPCU. The ICU bed elasticity was defined by the method by Gooch.^7^

### Health Economic Data

Economic data modeling was designed on the basis of the ICU bed cost per day and published average cost per day, using an estimated cost savings on the basis of our previous work of $2,600 per patient, multiplied by the total number of patients admitted to the NPCU who bypassed the NSICU.^8^ Time-driven activity-based cost (TDABC) modeling was applied to this study, on the basis of the Harvard Business School methodology (Table 2)^9^, ^10^, ^11^; this model was used to analyze and compare the economic benefits of implementing the NPCU model.

**Table 2:** Step by Step Time-Driven Activity-Based Costing Analysis

Steps
1. Develop process maps with the following principles: a. Each step reflects an activity in patient care delivery. b. Identify the resources involved for the patient at each step. c. Identify any supplies (disposables) used for the patient at each step. 2. Obtain time estimates for each process step through interviews and observations. 3. Calculate the capacity cost rate (CCR)^a^ for each resource: $\text{CCR}\;\text{of}\;\text{Resource}\;\text{A}=\frac{\text{Expenses}\;\text{attributable}\;\text{to}\;\text{Resouce}\;\text{A}}{\text{Practical}\;\text{capacity}\;\text{of}\;\text{Resource}\;\text{A}}$ 4. Calculate the total direct costs (personnel, equipment, space, and supplies) of all the resources used over the cycle of care.

a Capacity cost rate is how much it costs, per hour or per minute, for a resource to be available for patient-related work.

### Statistical Analyses

Data is presented using measures of central tendency for continuous variables; categorical variables are used for frequencies. Microsoft Excel version 16.56 was used for analysis.

## Results

### Patient Characteristics

A total of 77 neurosurgical patients used this NPCU protocol at our institution. Twenty patients were excluded as they underwent different procedures other than craniotomy for brain tumor (ie, thrombectomy, aneurysm coiling, cranioplasty, and ventriculoperitoneal shunt). Therefore, a final 57 patients who underwent craniotomies over 15 months were analyzed. The NPCU utilization was monitored after the delta surge had passed and more ICU beds became available. Once the delta surge passed, our normal PACU was converted back to the normal PACU on the fourth floor, and the fifth floor PACU resumed gastrointestinal procedures.

Over a period of 3 months, from July 1, 2021 to October 31, 2021 (delta surge), 37 patients with intracranial tumors followed this NPCU protocol: 23 (62.1%) were women, and the mean age was 57.7 years (range 35 to 83). Three different surgeons performed 37 craniotomies (Table 3); 22 (59.5%) were intervened for intra-axial lesions and 15 (40.5%) for extra-axial lesions. The most common type of intervention and etiology was craniotomy for meningioma resection (14 cases, 37.8.%). Thirty (81%) patients received general anesthesia, whereas 7 (19%) patients underwent procedures with monitored anesthesia care (conscious sedation).

**Table 3:** Patient Demographic Characteristics and Clinical Outcomes

Characteristic	Combined Cohort (n=57)	Delta Surge Cohort (n=37)	Post-Delta Surge Cohort (n=20)
Study length	15 mo	3 mo	12 mo
Number of patients (n)	57	37	20
Craniotomies	57	37	20
Diagnoses			
Meningioma	20 (35%)	14 (37.8%)	6 (30%)
Glioblastoma	10 (17.6%)	9 (24.4%)	1 (5%)
Metastatic lesions	7 (12.4%)	3 (8.1%)	4 (20%)
WHO grade 1 glioma	3 (5.3%)	1 (2.7%)	2 (10%)
Nondiagnostic biopsy	3 (5.3%)	3 (8.1%)	0
Radiation therapy changes	2 (3.6%)	1 (2.7%)	1 (5%)
WHO grade 2 glioma	2 (3.6%)	1 (2.7%)	1 (5%)
Gliosarcoma	2 (3.6%)	1 (2.7%)	1 (5%)
WHO grade 4 astrocytoma	1 (1.7%)	0	1 (5%)
Diffuse large B cell lymphoma	1 (1.7%)	1 (2.7%)	0
Cavernous malformation	1 (1.7%)	1 (2.7%)	0
Hemangiopericytoma	1 (1.7%)	1 (2.7%)	0
Subependymoma	1 (1.7%)	1 (2.7%)	0
Granulomatous inflammation	1 (1.7%)	0	1 (5%)
Subdural fibrous membrane	1 (1.7%)	0	1 (5%)
Subdural hematoma	1 (1.7%)	0	1 (5%)
Mean age (y) (range)	56.31 (25-83)	57.7 (35-83)	53.7 (25-81)
Sex			
Man	23 (40.4%)	14 (37.9%)	9 (45%)
Woman	34 (59.6%)	23 (62.1%)	11 (55%)
Patients transferred to NPCU	55 (96.5%)	35 (94.6%)	20 (100%)
Patients with good clinical outcome	55 (100%)	35 (100%)	20 (100%)
Mean ± SD POD at discharge	1.45±0.81	1.54±0.95	1.3±0.47
Patients transferred to NSICU	2 (3.5%)	2 (5.4%)	0
Patients with good clinical outcome	2 (100%)	2 (100%)	
Mean POD at discharge	4	4	

NSICU, neurosciences intensive care unit; NPCU, neurosciences progressive care unit; VP, ventriculoperitoneal; WHO, World Health Organization.

After the COVID-19 delta wave, the institution continued to admit patients to NPCU under the same protocol from November 1, 2021 to September 30, 2022. Twenty patients were admitted, 19 for intracranial tumor resection and 1 for subdural hematoma evacuation: 11 (55%) were women, and the mean age was 53.7 years (range 25 to 81). Three different surgeons performed 20 craniotomies (Table 3); 12 (60%) were intervened for intra-axial lesions and 8 (40%) for extra-axial lesions. The most common type of intervention and etiology was craniotomy for meningioma resection (6 cases, 30%). Nineteen (95%) patients received general anesthesia, and 1 (5%) patient received monitored anesthesia care (conscious sedation).

### Clinical Outcomes and Safety

All patients were taken to PACU after surgical intervention and were evaluated for transfer to NPCU as recommended by the treating surgeon preoperatively. After clinical assessment and application of the checklist-criteria (Table 1), 55 (96.5%) patients were transferred to the NPCU, and 2 (3.5%) patients were sent to the NSICU. These 2 patients required pain or vasopressor management after intra-axial tumor resection (Table 4) but had no unexpected clinical issues or untoward outcomes, all of them in the delta surge cohort. All 55 (100%) patients transferred to NPCU had good (expected postoperative) outcomes with no reported complications and none required transfer to NSICU or readmission after discharge. The mean postoperative day at discharge was 1.45 (SD±0.81) days with a range of 1 to 6 days.

**Table 4:** Patients Transferred to NSICU After Evaluation in PACU

Age (y)	Sex	Procedure	Diagnosis	Reason
52	Woman	Awake craniotomy	Glioblastoma	Anxiety and pain management
46	Man	Asleep craniotomy	Subependymoma	Vasopressor management

NSICU, neurosciences intensive care unit; PACU, postoperative care unit.

### Health Economic Data

**Figure 2:**
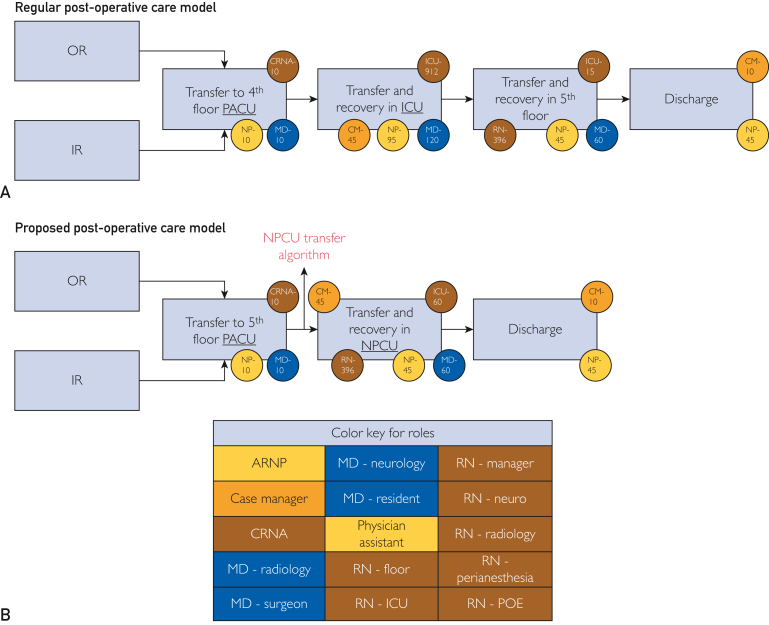
Time-driven activity-based cost (TDABC) maps for patients undergoing neurologic operation through ICU and NPCU tracks. (A) Regular postoperative care model pathway showing PACU and ICU admissions immediately after operation. (B) Proposed postoperative care model pathway showing PACU and NPCU admissions after PACU checklist-criteria application. Staff and time spent on each task is identified by the color circles; abbreviations and color key can be found in the 3×5 table. ARNP, advanced registered nurse practitioner; CM, case manager; CRNA, certified registered nurse anesthetist; IR, interventional radiology; MD, medical doctor; NPCU, neurosciences progressive care unit; PACU, postoperative care unit; OR, operative room; POE, postoperative evaluation; RN, registered nurse.

Fifty-five patients were transferred to the NPCU after PACU initial monitoring and NPCU readiness criteria were met. An estimate of cost savings was extrapolated from the total number of NPCU patients multiplied by our previous TDABC work of $2600 per patient because it was estimated that every patient transferred to the NPCU freed up 1 ICU bed for other critically ill surgical or medical patients. The estimated cost savings for this volume of patients and ICU beds saved was approximately $143,000 over the 15-month period. The TDABC maps are shown in Figure 2. Indirect cost savings and other downstream savings of freeing 55 ICU beds for other urgent or emergent ICU level surgical cases and extracorporeal membrane oxygenation (ECMO) COVID-19 patients was not performed.

## Discussion

The COVID-19 pandemic was associated with at least 80 million infections and more than 1 million deaths in the United States.^12^ Three years since the first COVID-19 case was reported in the country, multiple infection waves caused several hospital bed capacity challenges that further affected patient care across all disciplines in medicine and surgery. Health care institutions have tried to adapt to these unprecedented circumstances by modifying workflows and adapting hospital areas to accommodate the increased patient volumes.^13^^,^^14^

Our experience with COVID-19 infection waves prompted us to design this overflow workflow that could help neurology or neurosurgery patients in need of urgent or emergent operation from future COVID-19 variants and similar surges in hospital admissions. Historically, neurosurgery craniotomy patients are transferred to the NSICU for close neurological and vital sign monitoring. We therefore selected a subset of neurosurgery patients who had a low risk of deterioration for this model but were managed by the same neurocritical care team postoperatively but on a different floor. The ability to build this pathway for these neurosurgery cases also helped our fourth floor ICU accommodate patients with severe COVID-19 acute respiratory distress syndrome in need of ECMO. In our proposed workflow, 2 different assessments were used in 2 different settings to increase the accuracy of patient selection. First, the treating surgeon decides which patients may be suitable for NPCU transfer before surgical intervention on the basis of patient’s age, comorbidities, diagnosis, and clinical status. Second, after operation, the patient is transferred to PACU where they are observed for a few hours and assessed with the checklist-criteria to evaluate stability to transfer to NPCU.

The neurosurgeon’s a priori impression or pretest probability of needing ICU level of care is an important characteristic in this model because it served as the fundamental branch heuristic or algorithm to approach patients suitable for NPCU admission. All patients (100%) in this model that met the initial 2 branch points (ie, treating surgeon and PACU team appropriateness criteria) ultimately got discharged with a mean postoperative day of 1.45 with no long-term complications from this minor model change. This dynamic readiness or appropriateness criteria functions at 2 critical timepoints and involves multiple care team members as safeguards. This model therefore allowed the teams to reassess patients intraoperatively for changes in NPCU appropriateness and within the PACU phase. This allows further tailoring of the final bed status for either the NSICU or, if appropriate, to the NPCU or intermediate care unit.

Our results appear to be in accordance with previously reported analysis on ICU-need screening after craniotomy procedures.^15^ Young et al^16^ studied 3 types of interventions including tumor resection, microvascular decompression, and Chiari decompression; even though they do not describe subsequent reassessment after an initial screening process by the treating surgeon, none of the patients were upgraded to ICU and an estimated $422,128 direct cost were saved in 94 patients. In another study by Florman et al,^17^ inclusion and exclusion criteria were implemented preoperatively and postoperatively, although 5 patients out of 200 had to be upgraded to ICU services; all patients underwent craniotomies for tumor resection and the estimated cost savings were not reported.

Our initial concern before embarking on this model was potential complications of postoperative seizures, intracranial bleeding, and sudden neurological deterioration. Because these patients were super-selected and chosen by the treating neurosurgeon or anesthesia team, they likely represented fewer complex craniotomy patients (42 craniotomy patients were admitted to NSICU during delta wave, whereas 35 were followed in NPCU representing 45% of these cases). We also acknowledge excellence in neurology nursing monitoring to detect these complications because it would have triggered a change in the type of bed status needed with our NSICU team. Even though we did not find any major complications from this model, we acknowledge that interprofessional team communication and judgment between teams are paramount (ie, neurosurgeon to anesthesia, OR/PACU/NSICU/NPCU nursing, and neurosurgeon or anesthesia to NSICU teams).

Advances in neurosurgical techniques such as awake craniotomy programs and newer anesthetic agents may provide future opportunities to facilitate recovery and reduce ICU or hospital length of stay. The increase in popularity of regional anesthetic techniques such as scalp block and awake craniotomies, are showing promising results to reduce postoperative pain, duration of mechanical ventilation, postoperative nausea, and somnolence in our practice.^18^ Innovations in intraoperative seizure monitoring^19^ and awake cerebral mapping,^20^^,^^21^ have also emerged in our neurosurgical practice, which allows a better postoperative examination in the operating room and PACU. This leaves fewer patients left intubated to emerge and extubate in the PACU as a result. We acknowledge this as being a particular subset of patients in this cohort.

These trends beg the question of whether NSICU should be standard of care for all neurosurgical craniotomy patients. We believe that 1 size does not fit all patients and there is a role for individualized medicine approach in preoperative and intraoperative selection for downstream bed status and monitoring (and resources). Lessons we learned during our COVID-19 critical hospital census issues with this model may not be applicable to other hospitals and resources. Furthermore, this model might be better called an intermediate ICU for future neurosurgery patients rather than a PCU. We are not certain that the term progressive care unit is the correct term for this model after reviewing most of these cases because most of them meet the NSICU level of care at most other centers. Many of the services provided in the ICU include frequent neurological and vital sign examinations, and electrocardiographic monitoring, among others. Individualizing each case beginning with the preoperative assessment can allow a more tailored postoperative plan and avoids a 1-size-fits-all approach. Far beyond the financial cost savings, these 55 ICU beds that were freed within our ICU allowed for the care of other critically ill patients in need of mechanical ventilation, ECMO, or surgical critical care services and emergent operation.

### Study Strengths, Limitations, and Future Directions

To the best of our knowledge, this is one of the first studies addressing NSICU surge capacity and ICU bed elasticity modeling specifically for the neurology or neurosurgery ICU practice needs. This novel neurosurgical workflow and postoperative care pathway proved to be effective at identifying patients suitable for transfer to the NPCU, with no major complications to the patients. There are several major limitations of this study, which include the relatively small number of patients involved for analysis. However, these data represent an overflow model that other neurosurgical high-complexity and NSICUs may consider during tight bed census issues or to help during periods that can help drive growth to build larger NSICU bed systems.

We also acknowledge that neurology nursing is a critical resource in this model and that our neurology or neurosurgical floor nursing and their expertise allowed this model to exist within the added infrastructure of electronic ICU monitoring, and the extension of our NSICU team rounding and responding to these patients. Not all centers and hospitals have the required neurology nursing to make this model possible to detect and capture events that trigger immediate intervention. For example, the nurse staffing ratios required for this were intermediate high intensity staffing or q1-hour neurological checks × 12 to 24 hours before downgrading this intensity to floor nursing-to-patient ratios. This did increase our nursing-to-patient ratios temporarily for this NPCU before downgrade returned it toward floor level staffing ratios, in most cases 12 to 24 hours later. The initial triage performed at the discretion of the clinician-neurosurgeon limits the translation capacity for checklist-criteria at other institutions; however, given the dynamic factors intraoperatively, we felt the surgeon’s insight would be critical for this NPCU concept.

Finally, we acknowledge limitations of this model beyond the COVID-19 surge months and whether this is sustainable long-term model vs purely a Mobile Army Surgical Hospital-like unit temporarily. There are numerous challenges maintaining this model after the surge receded and return to business as usual at our hospital. Therefore, efforts were made to reduce stress and strain on our nursing staff on the fifth floor of the NPCU. Our NSICU team on the fourth floor acknowledged that it was easier for them to receive these craniotomy cases in the NSICU given the many years of experience and nurse training with these patients and the staffing ratios in the ICU proper (2:1). Therefore, long-term, our team has kept this model active for overflow situations for our NSICU capacity and to help us grow in the future to build a larger NSICU dedicated unit. We are considering creation of a hybrid NSICU-ICU, wherein the intermediate ICU represents an intermediate level of acuity because these patients have a higher turnover rate and a shorter length of stay in general. In fact, other NSICU academic centers use a similar model within their NSICU beds and by the time 1 postoperative case is discharged the next operating room neurosurgical case arrives in the NSICU. We acknowledge that this model is very efficient and may have different names at other academic and private NSICUs with the same goal in mind for providing high-quality, high efficiency patient care for neurosurgical ICU patients.

## Conclusion

Our preliminary findings suggest early safety and feasibility of preselected lower-risk neurosurgical cases for NPCU intermediate level of care during critical ICU bed census issues. A flexible, adaptable ICU or PCU bed staffing model allows a small amount of bed elasticity to allow other medical or surgical patients to receive ICU and complex ICU bed needs (ECMO, cardiothoracic surgery, and transplant).

## Potential Competing Interests

The authors report no competing interests.

## Supplemental Online Material

Strobe
